# Supplementary figures and images for: Development and validation of the facial scale (FaceSed) to evaluate sedation in horses
Source: PLoS One. 2021 Jun 1;16(6):e0251909. doi: 10.1371/journal.pone.0251909 (PMC8168851; doi:10.1371/journal.pone.0251909)

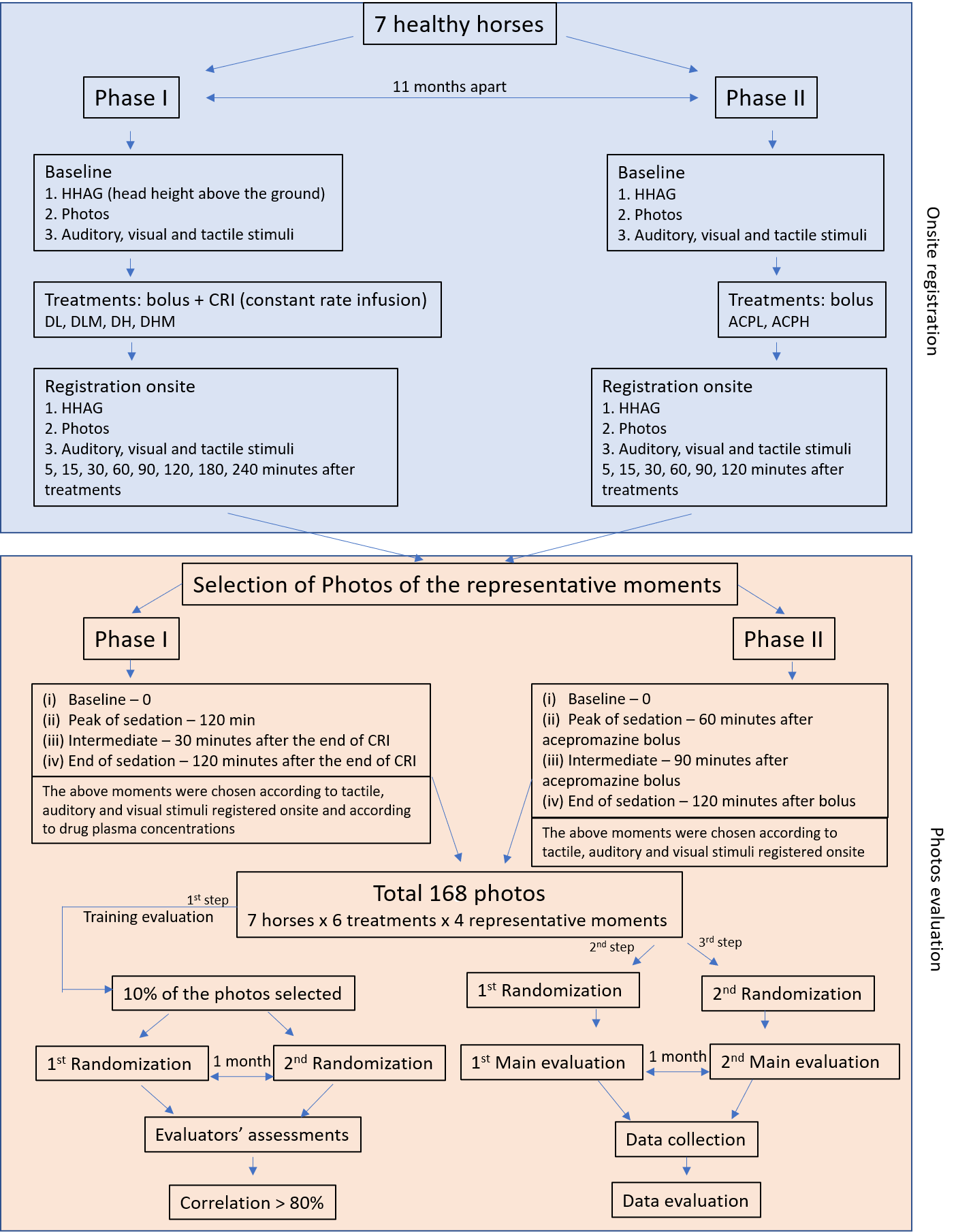

Supplement: S1 Appendix — (TIF) [file pone.0251909.s001.tif]
