# Supplementary material for: Development and validation of the facial scale (FaceSed) to evaluate sedation in horses
Source: PLoS One. 2021 Jun 1;16(6):e0251909. doi: 10.1371/journal.pone.0251909 (PMC8168851; doi:10.1371/journal.pone.0251909)
Supplement: S2 Appendix — (DOCX) [file pone.0251909.s002.docx]

**S2 Appendix. Guidelines for evaluation of a facial sedation scale in horses.**

Dear Evaluator,

Please read carefully the following instructions before you start.

Explanation of the photos:

Please open the file “1st Randomization - main analysis - photos”.

Each file of these photos have two photos of the same animal at the same moment and both must be seen to apply the facial scale. These photos were taken at the baseline and after treatments corresponded to the following situations: before sedation, intermediate and deep sedation and last moment when sedation was abated.

The files sent in word document attached to be filled for each step of the evaluation consist of different Scales: 1. The “**Numerical Scale**”, consisting of 0 for no sedation and 10 for the maximum possible sedation; 2. The “**Sedation Scale (FaceSed)**” to fill the score of each variable, according to the scale presented in the other word document attached (training word file).

The analysis is described in steps:

- Familiarization of the scales
- 1^st^ training
- 2^nd^ training
- 1^st^ part of the main evaluation
- 2^nd^ part of the main evaluation

1. **Familiarization**: Please read the scale before starting the analysis and if you have any doubts, suggestions or concerns, please contact us before starting the analysis. This will take about one hour of your time.

Steps **2** and **3** are important to guarantee a good intra-observer confiability

**2. 1^st^ training**: The will be shared with you on google drive:

“1^st^ Randomization – training photos” file with the photos, including 16 files and numbered as “1, 2, 3, 4 … 16”.

Each file with two photos, a total 32 photos should be analysed.

This analysis will take about two hours of your time.

Deadline: _____

After that, once you receive the new lot of photos, you should follow the instructions below.

**3. 2^nd^ training:** The file will be shared with you on google drive:

“2^nd^ Randomization – training photos” file with the photos, including 16 files and numbered as “1, 2, 3, 4 … 16”.

Each file with two photos, a total 32 photos should be analysed.

This analysis will take about two hours of your time.

Deadline: _____

After that, once you receive the new lot of photos, you should follow the instructions below.

**4.** **1^st^ part of the main evaluation:** The file will be shared with you on google drive:

1^st^ Randomization – main analysis - photos” file containing the photos numbered as “1, 2, 3, 4 … 168”.

This analysis will take about 8 hours of your time.

Deadline: **_____**

After that, once you receive the new lot of photos, you should follow the instructions below.

**5. 2^nd^ part of the main evaluation:** The file will be shared with you on google drive:

“2^nd^ Randomization – main analysis photos” file containing the photos numbered as “1, 2, 3, 4 … 168”.

This analysis will take about 8 hours of your time.

Deadline: _____

**6.** The scales must be filled in the order of the sheet in word document attached: 1. The **Numerical Scale**; 2. The **Sedation Scale (FaceSed)**.

**7.** We strongly recommend you should not perform the analysis longer than 1 hour a day, as the fatigue may compromise the quality of the analysis.

**8.** The beginning and deadline of each step is described in the table below. If it is not possible to follow the schedule, please let us know. It´s more important for us that the scales are filled carefully than complying the schedule.

**9.** After the conclusion of each phase, please send the word files to Alice Rodrigues de Oliveira ([aliceoliveira.br@gmail.com](mailto:aliceoliveira.br@gmail.com)).

Thank you for your contribution!

Best regards

Alice and Stelio

Table 1. Schedule

| **Steps** | **Start** | **Deadline** | **Action** | **Name of the file** | **Expected time** |
| --- | --- | --- | --- | --- | --- |
| Familiarization with the scale |  |  | Analysis of the photos exemplifying each item of the scale for doubts or concerns | Guidelines - photos | 1 hour |
| 1^st^ training |  |  | Analysis of 32 photos | 1^st^ Randomization – training photos | 2 hours |
| 2^nd^ training |  |  | Analysis of 32 photos | 2^nd^ Randomization – training photos | 2 hours |
| 1^st^ part of the main analysis |  |  | Analysis of 336 photos | 1^st^ Randomization – main analysis photos | 8 hours |
| 2^nd^ part of the main analysis |  |  | Analysis of 336 photos | 2^nd^ Randomization – main analysis photos | 8 hours |

Scales:

Numerical scale

| 0 | 1 | 2 | 3 | 4 | 5 | 6 | 7 | 8 | 9 | 10 |
| --- | --- | --- | --- | --- | --- | --- | --- | --- | --- | --- |

Deeply sedated

No sedation

| **Facial Sedation Scale** | | |
| --- | --- | --- |
| **Area evaluated** | **Relaxation Intensity** | **Scores** |
| **Ears** | No opening between the ear tips, position of attention | 0 |
|  | Partial opening between the ear tips or asymmetry | 1 |
|  | Wide opening between the ear tips (ears relaxed) | 2 |
| **Eyes (orbital opening)** | Eyes completely opened | 0 |
|  | Eyes partially opened | 1 |
|  | Eyes almost or completely closed | 2 |
| **Relaxation of the lower lip** | No signs of lower lip relaxation and/or closed mouth | 0 |
|  | Slight relaxation of lower lip | 1 |
|  | Pronounced relaxation of lower lip and/or open mouth | 2 |
| **Relaxation of the upper lip** | No signs of upper lip relaxation | 0 |
|  | Slight relaxation of upper lip | 1 |
|  | Pronounced relaxation of upper lip | 2 |
