# Supplementary material for: Development and validation of the facial scale (FaceSed) to evaluate sedation in horses
Source: PLoS One. 2021 Jun 1;16(6):e0251909. doi: 10.1371/journal.pone.0251909 (PMC8168851; doi:10.1371/journal.pone.0251909)
Supplement: Attachment — Submitted filename: Response to reviewers April 24th 2021.pdf [file pone.0251909.s005.pdf]

PONE-D-20-32183
Development and validation of the facial scale (Facesed) to evaluate sedation in horses

Reviewer #1: I re-read this study with a lot of interest once again. I congratulate the authors in carefully responding to all the comments and making changes accordingly. I believe the manuscript is now much more informative and clearer. Although it is a long read, it is a good example of a study that has enough detail for replication and for fully understanding what has been done. I have just a couple of minor comments now, please see below. And also a small note about line numbering in the highlighted document with the changes. I know this is a painstakingly process to get right (I always struggle myself whenever doing it in my own papers), but most of the lines mentioned in the answer are not the same as the highlighted manuscript. This made me take a little while longer to find all the changes, so it is something to keep in mind on future reviews. In any case, well done!

Answer:

Dear Reviewer

We appreciate your comments and thank you for your final suggestions.

Please find below each point answered separately.

Line 43-45: Explain briefly why is this a limitation. Is it one limitation or two? As in, using pictures AND horses were docile? (This might be explained in the text, but I was a bit confused when reading it here only)

Answer: included

Line 75: Do these scales require experience with the scale and/or horses? So only certain expert observers can use them? Is there any assessment of quality for how the observers use these scales (other than inter-observer reliability)?

Answer: These scales will require experience “with the effects of sedation in horses” (included). This point has also been addressed in the last sentence of the same paragraph “Unidimensional scales (VAS, NRS and SDS) may be biased to the interpretation and experience of the evaluator, generating differences in results with doubtful representativeness when comparing studies”.

While the clarifications and the author answers were all fantastic, I still struggled to follow the protocols and methodologies, particularly with the different phases and what belongs to what study. What about having a flow diagram to make it clear and easy to understand what was done, when, and for what studies?

Answer: a flow chart has been included as requested.
